# Integrated control of wood destroying basidiomycetes combining Cu-based wood preservatives and *Trichoderma* spp.

**DOI:** 10.1371/journal.pone.0174335

**Published:** 2017-04-05

**Authors:** Javier Ribera, Siegfried Fink, Maria del Carmen Bas, Francis W. M. R. Schwarze

**Affiliations:** 1 Applied Wood Materials, Empa, St. Gallen, Switzerland; 2 Professur für Forstbotanik, Albert-Ludwigs-Universität Freiburg, Freiburg im Breisgau, Germany; 3 Applied Statistics and Operational Research and Quality, Universitat Politècnica de València, València, Spain; USDA Forest Service, UNITED STATES

## Abstract

The production of new generation of wood preservatives (without addition of a co-biocide) in combination with an exchange of wood poles on identical sites with high fungal inoculum, has resulted in an increase of premature failures of wood utility poles in the last decades. Wood destroying basidiomycetes inhabiting sites where poles have been installed, have developed resistance against wood preservatives. The objective of the *in vitro* studies was to identify a *Trichoderma* spp. with a highly antagonistic potential against wood destroying basidiomycetes that is capable of colonizing Cu-rich environments. For this purpose, the activity of five *Trichoderma* spp. on Cu-rich medium was evaluated according to its growth and sporulation rates. The influence of the selected *Trichoderma* spp. on wood colonization and degradation by five wood destroying basidiomycetes was quantitatively analyzed by means of dry weight loss of wood specimens. Furthermore, the preventative effect of the selected *Trichoderma* spp. in combination with four Cu-based preservatives was also examined by mass loss and histological changes in the wood specimens. *Trichoderma harzianum* (T-720) was considered the biocontrol agent with higher antagonistic potential to colonize Cu-rich environments (up to 0.1% CuSO_4_ amended medium). *T*. *harzianum* demonstrated significant preventative effect on wood specimens against four wood destroying basidiomycetes. The combined effect of *T*. *harzianum* and Cu-based wood preservatives demonstrated that after 9 months incubation with two wood destroying basidiomycetes, wood specimens treated with 3.8 kg m^-3^ copper-chromium had weight losses between 55–65%, whereas containers previously treated with *T*. *harzianum* had significantly lower weight losses (0–25%). Histological studies on one of the wood destroying basidiomycetes revealed typical decomposition of wood cells by brown-rot fungi in Cu-impregnated samples, that were notably absent in wood specimens previously exposed to *T*. *harzianum*. It is concluded that carefully selected *Trichoderma* isolates can be used for integrated wood protection against a range of wood destroying basidiomycetes and may have potential for integrated wood protection in the field.

## Introduction

After many decades of use, wood remains the preferred choice for utility poles because of its availability, durability, strength-weight ratio and life cycle advantages. Copper (Cu) is the primary biocide component presently used to protect wood against soft-rot fungi in ground contact [1], but fails to protect against a range wood destroying basidiomycetes [2, 3]. Diverse mechanisms involved in the Cu-tolerance process of basidiomycetes have been described such as the ability to absorb Cu into cell wall components, extracellular chelation by metabolites and intracellular complexion with metallothioneins [4–9]. The absence of a co-biocide, in combination with an exchange of wood poles on identical sites with high fungal inoculum, has resulted in an increase in the number of premature failures of wood utility poles in the last decades [3, 10]. The economic damage due to premature failure of wood poles amounts to approx. 36 millions € per annum in Germany [3]. In a recent study by Ribera et al. [3], 73 utility poles of Norway spruce wood (*Picea abies* L.) impregnated with Cu-based wood preservatives from Switzerland and 38 from Germany and showing decay were selected and the associated wood destroying basidiomycetes isolated and analysed. The severity of decay differed according to the type of wood preservative formulation used and service life recorded. Brown-rot fungi and in particular *Antrodia* species were predominantly isolated from wood utility poles that were not treated with a co-biocide e.g. boron [3].

The search of environmentally friendly solutions to control undesirable microorganisms has resulted in great interest. Alternative management strategies are necessary to control fungi that have developed resistance to one or more pesticides. Integrated pest management is based on combining different control methods like cultural control, chemical control or biological control to reduce damage to crops and other commodities. The use of *Trichoderma* spp. as a biocontrol agent has been studied for decades [11–16]. A number of studies provide evidence that carefully selected *Trichoderma* spp. possess a high antagonistic activity against a range of wood destroying basidiomycetes [17–21]. The different mechanisms involved in the antagonistic process have been well described by several authors [17–21]. Although a combination of all mechanisms defines the antagonistic potential of each *Trichoderma* spp. it is believed that siderophores, volatile compounds and hydrolytic enzymes such as chitinases and glucanases play the most important role on the target hosts [19].

A commercial mixture of *Trichoderma polysporum* and *T*. *harzianum* was used in laboratory tests by Morrell and Sexton [22] to control decay of loblolly pine (*Pinus taeda* L.) sapwood and Douglas-fir (*Pseudotsuga menziesii* Mirb.) heartwood by five basidiomycetes. Although the product demonstrated a high antagonistic potential to protect wood against *Neolentinus lepideus* and *Rhodonia placenta*, most of the tested fungi were not completely inhibited. The selected *Trichoderma* spp. had little effect on white-rot fungi, or even on *N*. *lepideus*, when wood was exposed to soil in the laboratory. In another study, Bruce et al. [23] reported that wood cores extracted from utility poles colonized by *Trichoderma* were able to reduce decay by *N*. *lepideus* and *Antrodia carbonica* in laboratory studies. These are two of the most common basidiomycetes that decay pine (*Pinus palustris* Mill.) and Douglas-fir (*P*. *menziesii*) wood poles [23]. These results and the earlier findings by Highley and Richard [24] clearly show that wood blocks pre-treated with *Trichoderma* (Binab) were resistant to decay by brown-rot fungi but offered little protection against white-rot fungi in the laboratory. In subsequent studies, Brown et al. [25] demonstrated a significant effect of *Trichoderma* to protect wood stakes against soft-rot and basidiomycete decay in the field. This study also confirmed that the application of *Trichoderma* would have a better chance for success as a ground line treatment for wood poles because of the continual moisture environment [25]. However, the use of *Trichoderma* in combination with new generations of wood preservatives has been insufficiently studied in long-term field trials.

The objective of this study was to evaluate the potential of different *Trichoderma* spp. that can be used in combination with the new generation of Cu-based wood preservatives and to identify a competitive strain for integrated wood protection against a range wood destroying basidiomycetes.

## Materials and methods

### Growth rates and conidiogenesis of *Trichoderma* spp. on Cu-amended medium

The effect of Cu-amended medium was evaluated on the growth and sporulation of *Trichoderma* spp. (Table 1). Petri dishes containing 2% MEA (Malt extract agar, OXOID, Pratteln, Switzerland) and different concentrations of anhydrous CuSO_4_ (Sigma-Aldrich, Buchs, Switzerland) (0%, 0.025%, 0.05%, 0.075%, 0.1%, 0.5% and 1%) were inoculated in the centre with 100 μl of *Trichoderma* spp. conidia suspension adjusted to 10^6^ spores ml^-1^. The growth rate (mm day^-1^) was determined by colony diameter measurements after 10 days. To evaluate the influence of Cu-toxicity on conidiation rates of *Trichoderma* spp. the same concentrations of Cu-amended medium were evaluated. After 10 days, the produced conidia were collected, filtered with 5 μm pore filters and counted in a Neubauer improved chamber (Marienfeld, Germany). The yield of conidia was normalised to the surface of the fungal colony according to Gandía et al. [26]. For each experimental treatment (growth and conidiation rates), five Petri dishes for each *Trichoderma* spp. were performed.

**Table 1:** Fungal strains used in the present study.

Wood destroying basidiomycetes	Fungal ID	*Trichoderma* spp.	Fungal ID
*Antrodia serialis*	LT577949	*T*. *atroviride* Karsten (T-685)	FR178524
*Fibroporia vaillantii*	LT577950	*T*. *harzianum* (T-720)	LN881560
*Serpula himantioides*	LT577951	*T*. *harzianum* (T-721)	LN881561
*Gloeophyllum sepiarium*	LT577952	*T*. *atroviride* (T-722)	LN881562
*Rhodonia placenta*	Empa 45	*T*. *koningiopsis* (T-723)	LN881563
*Coniophora puteana*	Empa 62		
*Gloeophyllum trabeum*	Empa 100		
*Fomitopsis pinicola*	Empa 567		

### Preventative effect of *Trichoderma* spp. against wood destroying basidiomycetes

Interaction tests with wood block specimens of Scots pine (*Pinus sylvestris* L.) sapwood (2 x 2 x 8 cm; radial, tangential, longitudinal) were performed as described by Ribera et al. [27] with following modifications. For evaluation of the preventative effect of *Trichoderma* spp. against wood destroying basidiomycetes, 45 autoclavable plastic containers (WEZ, Oberentfelden, Switzerland. dimensions; 40L x 60W x 15H cm) with 1000 g of vermiculite and 15 g of Scots pine sawdust were used as a substrate for the cultivation of wood destroying basidiomycetes (Table 1). Each fungus was tested on three autoclavable containers as a biological replicate. Before testing, the moisture content (MC) and water holding capacity (WHC) of vermiculite was determined according to ENV 807 [28]. The amount of water needed to bring the substrate to 75% of its WHC was calculated and added to the vermiculite. After 8 weeks incubation with the five wood destroying basidiomycetes at 22 (±1)°C and 70% relative humidity (RH), the boxes were inoculated with either *T*. *harzianum* (T-720) or *T*. *atroviride* (T-685) (100 ml of 10^6^ spores ml^-1^). Three separate containers (biological replicates) for each wood decay basidiomycete were kept as controls. *T*. *harzianum* (T-720) was selected due to its high resistance on Cu-amended medium. *T*. *atroviride* (T-685) was selected as a bench mark fungus due its high antagonistic potential as described in previous studies [29, 30]. After 2 weeks incubation with *Trichoderma*, three wood specimens (3 repetitions) were sterilised with ethylene oxide and placed into each container. Three wood specimens were also placed into each control containers. Determination of the initial wood dry mass was calculated by oven drying (103°C) test specimens during 24h. After 12 weeks incubation, the specimens were removed, carefully cleaned with a brush, oven dried (103°C) and the mass loss recorded. In addition, the lethal effect of *Trichoderma* spp. was evaluated by re-isolating the wood destroying basidiomycetes from the substrate. Five subsamples of the infected substrate were selected randomly from the surface of each container and then placed into five different Petri dishes containing 20 ml of 2% MEA with 2 ml of thiabendazole dissolved in lactic acid (Merck, Darmstadt, Germany) (T-MEA). Petri dishes were incubated at 22 (±1)°C and 70% RH for 10 days. T-MEA is a selective medium for wood destroying basidiomycetes [31]. If the wood destroying basidiomycete failed to grow on T-MEA, the lethal effect of *Trichoderma* spp. was considered to be 100%.

### Test method for determining the protective effectiveness of wood preservatives against wood destroying basidiomycetes

Evaluation of the toxicity of Cu-based wood preservatives was performed according to the European Standard EN113 [32]. Wood specimens of Scots pine sapwood with standard dimensions (2.5R x 1.5T x 5L cm) were impregnated using a vacuum-pressure process. Four concentrations of wood preservatives distributed close to the expected toxic value were selected and the retention rates of the wood specimens were calculated (Table 2). Specimens impregnated with distilled water (0% preservative formulation) were used as controls. Wood specimens were kept in a drying vessel during four weeks and inverted twice per week. Culture vessels were inoculated with *R*. *placenta*, *C*. *puteana*, *G*. *trabeum* and *F*. *pinicola* from the Empa collection (Table 1). In each culture vessel one Cu-treated wood specimen and one non-impregnated control specimen was placed. For each different amount of retention rates of wood preservative and the respective fungi, four biological replicates were used. After 16 weeks mass loss was recorded (as described above) and toxic values were calculated. The toxic thresholds were calculated for each wood preservative formulation at which the wood was not adequately protected against the wood destroying fungi. The protection provided for the wood by the test preservative was considered to be adequate when the mean loss in mass of the specimens was less than 3.0% of their initial dry mass.

**Table 2:** Cu-based wood preservatives retained by the wood specimens (EN113).

CCB* (kg m^-3^)	CC** (kg m^-3^)	Cr-free 1*** (kg m^-3^)	Cr-free 2*** (kg m^-3^)
40.9	36.7	26.1	43.3
26.7	21.1	20.3	22.6
18.0	13.3	8.7	15.7
8.7	7.1	4.6	5.5
0.0	0.0	0.0	0.0

*CCB = copper-chromium-boron;

**CC = copper-chromium;

***Cr-free = chromium free.

### Combined effect of Cu-based wood preservatives and *Trichoderma* spp. on wood destroying basidiomycetes

Wood stakes of Scots pine sapwood (2R x 2T x 8L cm) were impregnated with vacuum-pressure according to EN252 [33] with two different concentrations selected from the results obtained in the “Test method for determining the protective effectiveness of wood preservatives against wood destroying basidiomycetes”. One concentration below the toxic value was used to evaluate the integrated control effect of *Trichoderma* spp. as a biocontrol agent. The second concentration above the toxic value was used as a positive control for each wood preservative. Wood specimens impregnated with distilled water (0% preservative formulation) were used as controls. After impregnation, stakes were dried for four weeks to allow fixation of the wood preservatives.

Autoclavable containers containing vermiculite/sawdust (as described above) were inoculated with a range wood destroying basidiomycetes. *Antrodia serialis* was selected as it is the most common fungus isolated from infected wood utility poles in Switzerland and Germany [3]. *Serpula himantioides* was selected due to its strong decomposition rates observed in 0.1% Cu-treated wood (higher than 5% mass loss after 8 weeks) [3]. *R*. *placenta* (EMPA 45) was used as a positive control because of its high Cu-tolerance as demonstrated by Civardi et al. [2] and Ribera et al. [3]. In total, nine containers per wood destroying basidiomycete were prepared (three biological repetitions per fungal treatment). After 8 weeks, three containers (biological replicates) per wood destroying basidiomycete were inoculated with spore suspensions of *T*. *harzianum* (T-720) or *T*. *atroviride* (T-685) as described above. Three separate containers (biological replicates) for each wood decay basidiomycete were kept as controls. Two weeks after the *Trichoderma* treatment, three sterilised wood specimens from each Cu-based wood preservative were randomly placed into each container. In total 27 samples per box were incubated.

After 9 months incubation, the wood specimens were removed and the mass loss was recorded as described above. In addition, the lethal effect of *Trichoderma* was also evaluated.

### Light microscopy

For light microscopy, the incubated wood was cut into smaller blocks (1 × 0.5 × 0.5 cm), embedded in a methacrylate medium and subsequently polymerized at 50°C. The embedded specimens were sectioned at 3 μm using a rotary microtome (Leica^®^ 2040 Supercut) fitted with a diamond knife. The selected transverse sections were stained for 12 h in safranine and then counterstained for 3 min in methylene blue and 30 min in auramine [34]. Colour micrographs (Kodak EPY 64T) were taken with a Leitz Orthoplan microscope fitted with a Leitz-Vario-Orthomat camera system. Early stages of brown-rot (seen as loss of birefringence due to cellulolysis) were detected by viewing sections between crossed Nicols [35–38].

### Statistical analysis

Several statistical tests were performed with different objectives applying Analysis of Variance (ANOVA) to the growth, sporulation rates and wood mass losses variables. The normality assumption of the distribution data was checked via the Kolmogorov-Smirnov test. The *p*-values obtained rejected the normality at a 95% confidence level for growth measurements, sporulation rates and wood mass losses. Therefore, and following previous studies by Civardi et al. [2], a logarithmic transformation for growth and sporulation data and an arcsine transformation for wood weight losses were applied to obtain normality. All data was back-transformed to numerical values for presentation (expressed as mean ± SD). The ANOVA analysis was applied to assess the effect of Cu-amended medium on the growth and sporulation of each *Trichoderma* spp., considering measurements obtained from each Petri dish as biological replicates. For this purpose, Dunnett’s test was used to evaluate the differences in growth and sporulation between each treatment with CuSO_4_ to the control group (0% CuSO_4_) (significance levels of *p*<0.05). To compare the influence of Cu-amended medium on growth and sporulation between the *Trichoderma* spp., a Tukey’s HSD (Honestly Significant Difference) test was additionally performed for each concentration of CuSO_4_ (*p*<0.05).

To evaluate the preventative effect of *Trichoderma* spp. (T-720 and T-685) against each wood destroying basidiomycetes another Tukey’s HSD test was also performed. The wood specimens from each container were considered as repetitions. Statistics were then performed on the average of the three repetitions that was considered the value for each biological replicate. All the statistical analysis were performed using the statistical software SPSS^®^ (Version 22, SPSS Inc., Chicago, IL, USA).

## Results and discussion

### Growth rates and conidiogenesis of *Trichoderma* spp. in Cu-amended medium

Due to the potential application of *Trichoderma* in soils with a high content of leached preservatives around the wood poles [39–42] it was important to identify a competitive strain that was able to colonise Cu-rich substrates. Some authors have described a high Cu-tolerance of *Trichoderma* spp. to a wide range of Cu concentrations [43–49]. In this study, the toxic effect of CuSO_4_ amended medium on the growth of *Trichoderma* spp., compared by the Dunnett’s test, was statistically significant in concentrations above 0.1% CuSO_4_ for all strains (Table 3). The Tukey’s test showed significant differences between the growths of *Trichoderma* spp. in some concentrations of CuSO_4_. *T*. *atroviride* (T-722) demonstrated rapid colonization of the 0% Cu-amended medium compared to the other *Trichoderma* spp. However, T-722 was the most susceptible strain to CuSO_4_, being the only strain that ceased to grow at 0.075% (Table 3). *T*. *harzianum* (T-720) and *T*. *koningiopsis* (T-723) were highly tolerant to CuSO_4_-amended medium growing albeit more slowly in concentrations up to 0.75% of CuSO_4_.

**Table 3:** Effect of Cu-amended medium on the diametric growth of *Trichoderma* spp. after 10 days incubation on Petri dishes. Data represented as mean of five biological replicates (mm day^-1^ ± SD).

	0%	0.025%	0.05%	0.075%	0.1%	0.5%	1%
**T-685**	17.8(a) ± 0.1	18.8(a) ± 0.6	3.5(ab) ± 2.0	1.0(ab)* ± 0.7	0.0(a)* ± 0.0	0.0(a)* ± 0.0	0.0(a)* ± 0.0
**T-720**	18.3(a) ± 0.1	19.1(a) ± 0.2	3.7(ab)* ± 0.4	3.3(bc)* ± 0.3	3.5(b)* ± 0.7	0.0(a)* ± 0.0	0.0(a)* ± 0.0
**T-721**	19.6(a) ± 0.5	19.6(a) ± 0.7	9.1(a)* ± 0.0	4.8(c)* ± 0.2	0.0(a)* ± 0.0	0.0(a)* ± 0.0	0.0(a)* ± 0.0
**T-722**	23.0(b) ± 0.3	13.4(a) ± 0.4	1.4(b)* ± 0.7	0.0(a)* ± 0.0	0.0(a)* ± 0.0	0.0(a)* ± 0.0	0.0(a)* ± 0.0
**T-723**	19.5(a) ± 0.2	18.0(a) ± 0.2	5.1(ab)* ± 0.2	4.2(c)* ± 0.2	6.0(c)* ± 0.4	0.0(a)* ± 0.0	0.0(a)* ± 0.0

*Significant influence of growth by Cu-amended medium (Dunnett’s test: *p*<0.05).

Different letters denote significant differences between the fungi after the Tukey’s HSD test for each concentration of Cu-amended medium (column-wise).

Results of the Dunnett’s test to measure the effect of CuSO_4_ on the production of conidia showed that the effect was statistically significant (*p*<0.05) in concentrations above 0.075% CuSO_4,_ compared to the 0% CuSO_4_ for all *Trichoderma* spp. (Table 4). The Tukey’s comparisons between *Trichoderma* spp. did not show significant differences for concentrations above 0.1% CuSO_4_ since no *Trichoderma* spp. was able to colonise such a high concentrations of CuSO_4_ in the substrate. *T*. *harzianum* (T-720) produced higher concentrations of conidia in absence of CuSO_4_ (0% CuSO_4_). Moreover, T-720 was able to sporulate, albeit in reduced yield, in toxic concentrations of CuSO_4_ for the rest of the *Trichoderma* spp. (0% CuSO_4_). The toxic effect of CuSO_4_ on the production of conidia was more significant on the strains T-721 and T-722 compared to the other strains in 0.05% CuSO_4_, producing no conidia.

**Table 4:** Effect of Cu-amended medium on the conidiogenesis process of *Trichoderma* spp. after 10 days incubation on Petri dishes. Data represented as mean of five biological replicates (spore cm^-2^ ± SD).

	0%	0.025%	0.05%	0.075%	0.1%	0.5%	1%
**T-685**	7.94E^6^(a) ± 386	17.48E^6^(a) ± 386	42277(a)* ± 59757	0(a)* ± 0	0(a)** ± 0	0(a)* ± 0	0(a)* ± 0
**T-720**	44.53E^6^(ab) ± 887	35.42E^6^(a) ± 2530	5.96E^5^(a) ± 360	49052(b)* ± 40036	1293(b)* ± 1213	0(a)* ± 0	0(a)* ± 0
**T-721**	8.90E^6^(bc) ± 991	6.28E^6^(a)* ± 2672	0(b)* ± 0	0(a)* ± 0	0(a)** ± 0	0(a)* ± 0	0(a)* ± 0
**T-722**	16.68E^6^(bc) ± 2613	11.84E^6^(a) ± 9199	0(b)* ± 0	0(a)* ± 0	0(a)** ± 0	0(a)* ± 0	0(a)* ± 0
**T-723**	8.40E^6^(c) ± 67674	34.48E^6^(a) ± 51751	5.69E^6^(a) ± 1459	1.43E^6^(b) ± 4545	0(a)** ± 0	0(a)* ± 0	0(a)* ± 0

*Significant reduction of spore production by Cu-amended medium (Dunnett’s test: *p*<0.05).

Different letters denote significant differences between the fungi after the Tukey’s HSD test for each concentration of Cu-amended medium (column-wise).

### Preventative effect of *Trichoderma* spp. against wood destroying basidiomycetes

The diversity of results reported by different authors [22–25] emphasizes the importance to specifically screen a *Trichoderma* isolate with a high antagonistic potential against the target fungus. The present study indicates that careful selection of a *Trichoderma* isolate with bioassays will improve the success rate of the antagonist in the field.

**Fig 1:**
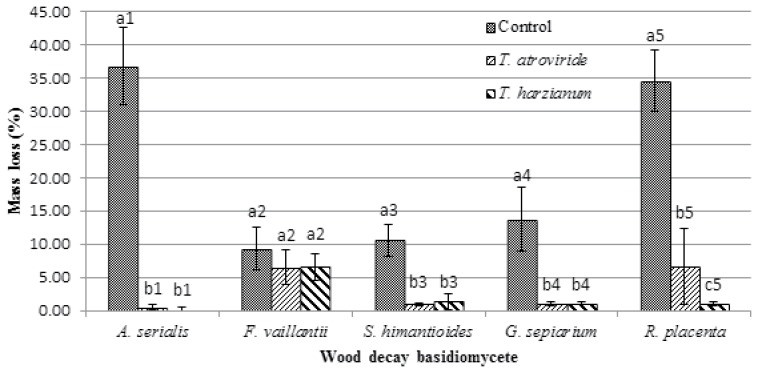
Effect of *Trichoderma* species, T-685 and T-720, on preventing mass loss from basidiomycetes after 12 weeks incubation on wood. Different letters indicate significant differences between the *Trichoderma* treatments and the controls. Shared numbers indicate the subgroups (wood destroying basidiomycete) of the analysis after the Tukey’s HDS test (*p*<0.05). Data represented as mean ± SD of three biological replicates.

Treatment with *Trichoderma* spp. significantly reduced mass losses of some wood specimens compared to the controls (Fig 1). *Trichoderma* spp. had a significant impact (*p*<0.05) on the decay process of four wood destroying basidiomycetes (*A*. *serialis*, *S*. *himantioides*, *G*. *sepiarium* and *R*. *placenta*). Even if *T*. *harzianum* (T-720) showed a higher preventative effect on the reduction of mass loss against *R*. *placenta*, Tukey’s test did not show significant differences between the effects of both *Trichoderma* spp. treatments against wood destroying basidiomycetes (Fig 1). Additionally, negative re-isolation of wood destroying basidiomycetes after 12 weeks from the wood specimens on selective medium (T-MEA), demonstrated a 100% lethal effect of the wood destroying basidiomycetes by the applied *Trichoderma* spp. (Table 5).

**Table 5:** Lethal effect of *Trichoderma* strains against Cu-tolerant basidiomycetes. Data represented as mean of five subsamples from three biological replicates.

	Controls	T-685 (%)	T-720 (%)
*A*. *serialis*	0 ± 0	100 ± 0	100 ± 0
*F*. *vaillantii*	0 ± 0	100 ± 0	100 ± 0
*S*. *himantioides*	0 ± 0	100 ± 0	100 ± 0
*G*. *sepiarium*	0 ± 0	100 ± 0	100 ± 0
*R*. *placenta*	0 ± 0	100 ± 0	100 ± 0

Different authors have previously reported the successful eradication of wood destroying basidiomycetes with biocontrol agents on wood poles. For instance, Ricard [50] described the use of *Scytalidium* spp., *Ascocoryne sarcoides* and *Trichoderma* to suppress decay by *N*. *lepideus* and *A*. *carbonica* in the field. By contrast, Bruce and King [51, 52] showed that successfully eradication of *L*. *lepideus* only appeared possible during very early stage of wood decay in the field. The latter preventative and remedial studies demonstrated difficulties of *Trichoderma* to develop and dominate highly infected creosote wood poles in the field. The versatility of *Trichoderma* spp. to grow on a wide range of acid and basic substrates as well as its tolerance to high concentrations of CO_2_ and N increases its potential as integrated control of wood destroying basidiomycetes as described by Score and Palfreyman [53].

### Determination of the toxic values against wood destroying basidiomycetes

**Fig 2:**
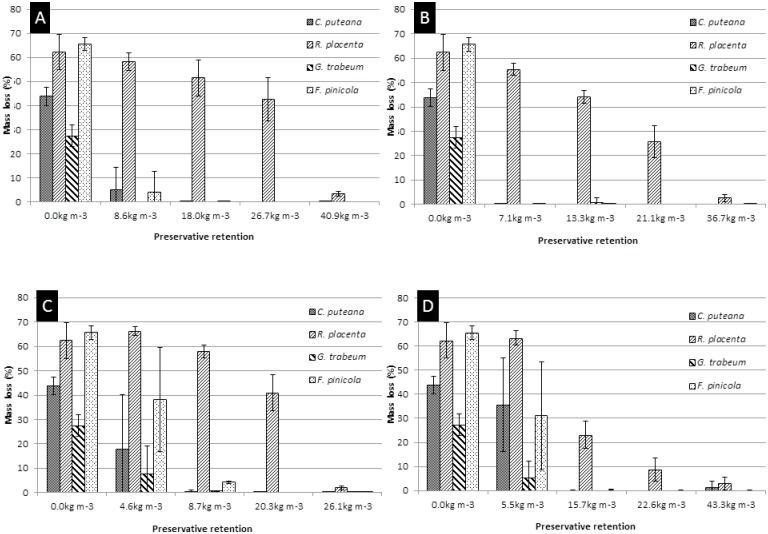
Results of wood mass loss according to EN113 (1996) after 16 weeks of fungal incubation. (A) CCB formulation. (B)CC formulation. (C) Cr-free formulation 1. (D) Cr-free formulation 2.

All selected concentrations were in the range of the expected toxic values for the fungi used in the EN113 test because all fungi reported mass loss of the specimens less than 3.0% of their initial dry mass for the highest amount of retention of wood preservative (Fig 2). The mass losses reported for the controls (higher than 20%) confirmed the minimum activity of the fungi required for the test. *R*. *placenta* (Empa 45) showed the highest Cu-tolerance for all Cu-based preservatives compared to the other fungi. The toxic effect of the Cu-based preservatives on *R*. *placenta* was used to select the toxic retention threshold for the integrated wood protection of *T*. *harzianum* against the wood destroying basidiomycetes. The non-toxic retentions were defined according to the minimum concentrations at which the wood was not adequately protected for *R*. *placenta* and at which other fungi could cause wood decay. The non-toxic value for the CC-treatment was chosen to be 50% of the minimum tested retention to guarantee the non-toxic values.

### Combined effect of Cu-based wood preservatives and *Trichoderma* spp. on wood destroying basidiomycetes

**Fig 3:**
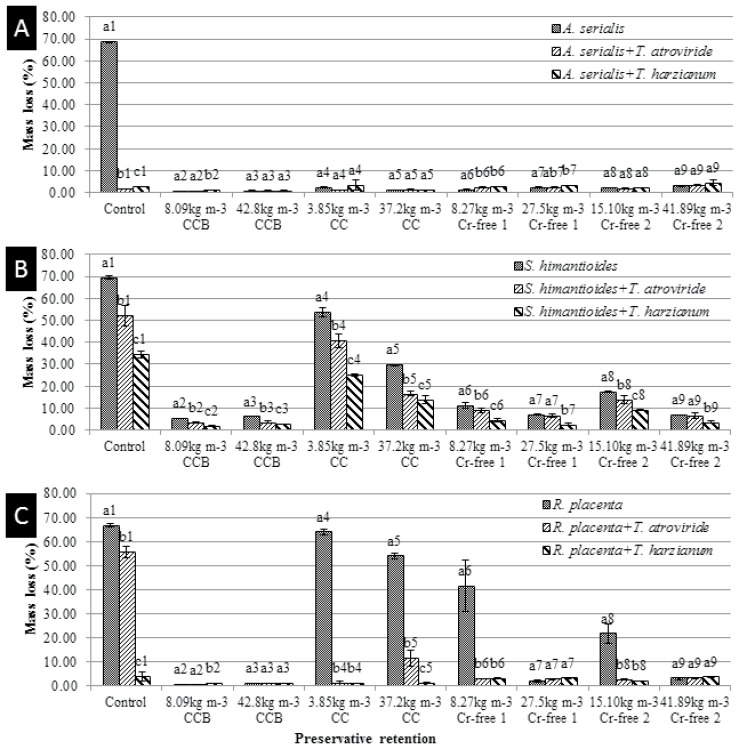
Effect of *Trichoderma* species, T-685 and T-720, on preventing mass loss from basidiomycetes after 9 months incubation on Cu-treated wood. (A) *A*. *serialis*. (B) *S*. *himantioides*. (C) *R*. *placenta*. Different letters indicate significant differences between the *Trichoderma* treatments and the controls. Shared numbers indicate the subgroups (wood destroying basidiomycete) of the analysis after the Tukey’s HDS test (*p*<0.05). Data represented as mean ± SD of three replicates.

The compatibility of *T*. *harzianum* (T-720) with a range of common wood preservatives demonstrated here and the good performance against wood destroying basidiomycetes was evident (Fig 3). *A*. *serialis* did not demonstrate a high resistance to the wood preservative formulations evaluated because it did not cause mass losses higher than 3% of the initial mass (Fig 3a). Nevertheless, the mass loss recorded for the control (absence of Cu-based and *Trichoderma* treatment) demonstrated a high activity of the fungus (nearly 70% of the initial mass). Wood samples treated without Cu-based preservatives showed a significant reduction of mass loss for both *Trichoderma* treatments. Besides, the treatment with *T*. *atroviride* (T-685) was significantly more effective to prevent wood decay. However, Tukey’s results did not show a positive effect of the *Trichoderma* spp. treatment for any Cu-impregnated specimens. *S*. *himantioides* caused mass losses higher than 5% in the control for all retentions rates of wood preservative formulations (Fig 3b). The application of both *Trichoderma* spp. to prevent decay against *S*. *himantioides* resulted in a reduction of mass loss for most of the Cu-impregnated wood. The preventative effect against wood decay basidiomycetes was only absent for high retentions of Cr-free preservative formulations (27.5 kg m^3^ Cr-free 1 and 41.89 kg m^3^ Cr-free 2). *T*. *harzianum* (T-720) demonstrated higher significant effect to prevent wood decay for all Cu-based treatments (Fig 3b). *R*. *placenta* caused high mass losses in the wood specimens used as controls (higher than 20%), CC preservative (3.85 kg m^3^ and 37.2 kg m^3^ CC) and low retentions of Cr-free preservatives (8.27 kg m^3^ Cr-free 1 and 15.10 kg m^3^ Cr-free 2) (Fig 3c). The preventative effect of *Trichoderma* treatment on those wood specimens was significant for all samples. Additionally, Tukey’s results only showed significant differences between both *Trichoderma* spp. for Cu-untreated wood specimens and 37.2 kg m^3^ CC (Fig 3c).

Interestingly, the general trend of a treatment with *Trichoderma* spp. was more evident on wood specimens impregnated with low concentrations of Cu-based wood preservatives that were incubated with *S*. *himantioides* (8.09 kg m^3^ CCB, 3.85 kg m^3^ CC, 8.27 kg m^3^ Cr-free 1 and 15.10 kg m^3^ Cr-free 2) and *R*. *placenta* (3.85 kg m^3^ CC, 8.27 kg m^3^ Cr-free 1 and 15.10 kg m^3^ Cr-free 2), respectively (Fig 3b and 3c). *T*. *harzianum* (T-720) showed a slightly higher antagonistic potential to protect Cu-impregnated and non-impregnated wood specimens against *S*. *himatioides* and *R*. *placenta*. Failure to re-isolate the wood destroying basidiomycetes from the wood specimens demonstrated 100% lethal effect by *Trichoderma* spp. (Table 6).

**Table 6:** Lethal effect of *Trichoderma* strains against Cu-tolerant basidiomycetes. Data represented as mean of five subsamples from three biological replicates.

	Controls	T-685 (%)	T-720 (%)
*A*. *serialis*	0 ± 0	100 ± 0	100 ± 0
*S*. *himantioides*	0 ± 0	100 ± 0	100 ± 0
*R*. *placenta*	0 ± 0	100 ± 0	100 ± 0

**Fig 4:**
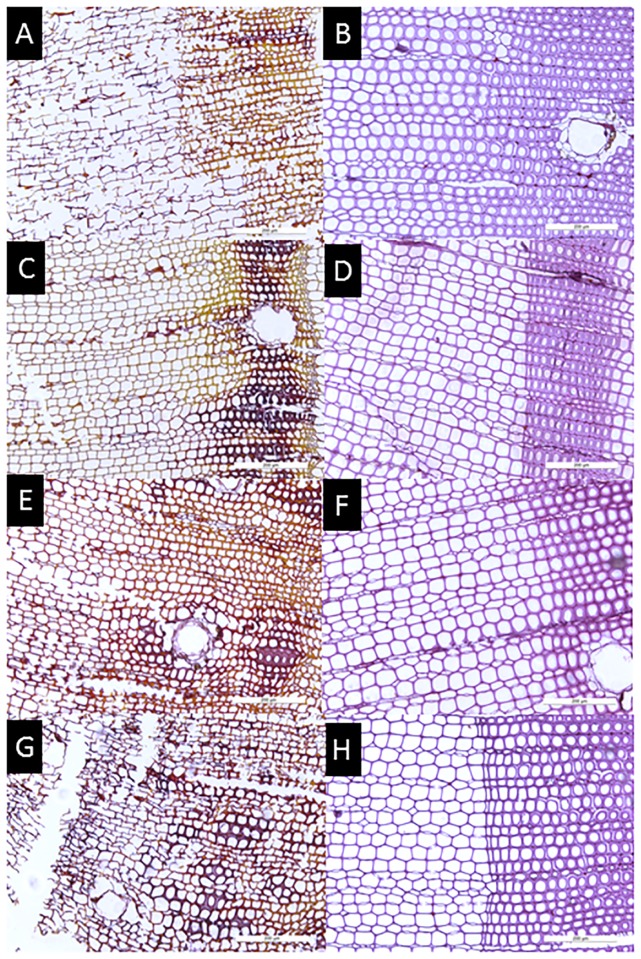
Combined effect of *Trichoderma* species, T-685 and T-720, on preventing mass loss from basidiomycetes after 9 months incubation on Cu-treated wood. (A) Control—*R*. *placenta*. (B) Control—*R*. *placenta* + *T*. *harzianum*. (C) 37.2 kg m^-3^ CC—*R*. *placenta*. (D) 37.2 kg m^-3^ CC—*R*. *placenta* + *T*. *harzianum*. (E) 8.2 kg m^-3^ Cr-free 1—*R*. *placenta*. (F) 8.2 kg m^-3^ Cr-free 1—*R*. *placenta* + *T*. *harzianum*. (G) 15.1 kg m^-3^ Cr-free 2—*R*. *placenta*. (H) 15.1 kg m^-3^ Cr-free 2—*R*. *placenta* + *T*. *harzianum*. (Bar 200μm, 10x).

The microscopic investigations demonstrated typical features of brown-rot i.e. numerous clefts in the secondary walls of the tracheids by *R*. *placenta* even when the wood was treated with wood preservatives (Fig 4c, 4e and 4g). Cracks appeared in the secondary wall extending perpendicular within the S2 from the S3 to the S1. The colour of degraded secondary walls changed from blue to red, indicating the preferential decomposition of hemicellulose and cellulose (Fig 4c, 4e and 4g). Under polarized light, secondary walls of early wood tracheids showed a distinct loss of birefringence, as also observed by Schwarze et al. [38]. Higher lignified parts, such as the late wood tracheids and epithelial cells of the resin ducts were more resistant to decay, as also described by Schwarze [54]. In contrast, low retentions of wood preservatives in combination with *T*. *harzianum* (T-720) were associated with an absence of features typically associated with brown-rot (Fig 4d, 4f and 4h).

Although previous studies could not demonstrate the protection of Cu-treated wood against a range of wood destroying basidiomycetes [55, 56], the combined use of *Trichoderma* spp. as a biocontrol agent against two wood destroying basidiomycetes on creosote treated poles has been demonstrated in the past [50]. Thus, years after application of *Trichoderma* in the field, Bruce et al. [57–59] could demonstrate the viability of the *Trichoderma* treatment on the same site. The reduction of inoculum of wood destroying basidiomycetes demonstrated in the laboratory by *T*. *harzianum* (T-720), shows promising potential as a long term treatment for the field. This method of integrated wood protection could improve the efficacy of Cu-based new generation wood preservatives against wood destroying basidiomycetes on high risk sites in Switzerland and Germany.

## Conclusions

In this study we demonstrated the possibility of using *T*. *harzianum* (T-720) as a biocontrol agent against a range of wood destroying basidiomycetes. The high Cu-threshold demonstrated by *T*. *harzianum* (T-720) suggests that this isolate might have a potential for integrated wood protection against Cu-tolerant wood decay basidiomycetes in infested soil around wood poles. The application of *Trichoderma* spp. for eradicating a range of wood destroying basidiomycetes under laboratory conditions and preventing wood decay was demonstrated. In the future, the application of carefully selected *Trichoderma* spp. in combination with more environmentally friendly wood preservative formulations (new generation of wood preservatives) and lower concentrations could be possible with more laboratory and long-term field testing. If future studies show that this method of integrated wood protection is successful in the field, it would be possible to enhance wood durability and prolong the service life of wood products. The development of such a sustainable method against wood decay would also help to regain traditional markets (i.e. wood sticks in orchards, hop -and vine yards) in which alternative materials such galvanized steel are currently in use. Long-term field studies with stakes and wood utility poles are currently in progress which will help to examine the performance of the selected *T*. *harzianum* (T-720) under natural conditions.
